# Suicidal behaviors: Relationship with body mass index and serological indicators

**DOI:** 10.1192/j.eurpsy.2021.1573

**Published:** 2021-08-13

**Authors:** G. Esgalhado, I. Costa

**Affiliations:** Psychology And Education, University of Beira Interior, Covilha, Portugal

**Keywords:** Total cholesterol, Triglycerides, BMI, Suicidal behaviors.

## Abstract

**Introduction:**

Current research has demonstrated associations between variables of a biomedical nature with the presence of psychological indicators.

**Objectives:**

To analyze the relationship between levels of total cholesterol, triglycerides, and Body Mass Index (BMI) with suicidal behaviors, on a non-smoking sample, without women who take birth control pills and participants without depressive pathologyTo analyze the relationship between levels of total cholesterol, triglycerides, and Body Mass Index (BMI) with suicidal behaviors, on a non-smoking sample, without women who take birth control pills and participants without depressive pathology.

**Methods:**

We used a sociodemographic questionnaire and the Suicidal Behaviors Questionnaire - revised (SBQ-R) to evaluate the suicide ideation, suicide attempt and the probability of committing suicide. The sample is composed of 166 participants with ages between 18 and 89-years-old, 54.2% are men and 45.8% are women.

**Results:**

We observed a weak association between serological indicators with some components of suicidal behaviors. It is also observed that higher cholesterol levels are associated with a higher probability of suicide; normal BMI is related to an increase of suicidal ideation; and the age group of 41 to 89 years-old presents a higher probability of committing suicide.

**Conclusions:**

It is further concluded that age, gender, marital status, place of residence, education and professional status are significantly associated with suicidality. Yet, the influence of cholesterol, triglycerides, and BMI levels on suicide behaviors was not supported.

